# Sight

**Published:** 1886-04

**Authors:** 


### SIGHT.

The peculiarities of sight are generally owing to the unusual prominence or depression of the pupil of the eye. When this is excessive, it arises from external conformation, but when only slight it depends upon the greater or less proportion of an exceedingly minute quantity of fluid immediately behind the pupil, which may increas, or modify its prominence.

**Near Sight** invariably arises in early life. When it arises from external convexity of the pupil it is generally incurable either by time or treatment, and the only remedy for the defect is a concave glass. But when there is no apparent convexity of the eye, it may be concluded that the defect arises from an excess of the fluid above mentioned, which always diminishes more or less. In these cases glasses should be resorted to very sparingly, or not at all, as there is good hope that the vision may improve as years advance. Near-sightedness in children often leads to an impression that they are stupid and deficient in power of general observation, until the real character of the defect is discovered.

**Far Sight** scarcely ever occurs ln early life, but usually sets in about middle age. The peculiarty is, that though distant objects can be seen as in youth, near objects, print, etc., are dim. This arises from a gradual diminution of the fluid before mentioned, causing the sight to get worse and worse. A convex glass is generally an effectual remedy, requiring to be exchanged for one still more convex, at intervals, as the fluid goes on diminishing.

**Ophthalmia** is the name given to inflammation of the eyes. It arises in cases of weak eyes or excessive exposure to light, cold winds, dust and other foreign matter. In ordinary cases it is limited to the eyelids and to the skin covering the white of the eyes only, which become blood-shot. There is a sensation as if sand were in the eye, when it is not, with much heat. There is a thick, yellow, mucus secretion which, in drying, often glues up the eyes during the night. Mild cases should be treated with warm water only, or with fomentations of poppy heads, with attention to the general health, and care to avoid exposure to either heat or cold. If the case is severe or of long standing, medical advice should be obtained, as neglect may soon lead to permanent impairment or loss of sight.

**Anamalcule**.—Animalcule is the name given to animal life when the individuals are too small to be distinctly seen with the naked eye. It was formerly thought that mites of cheese were the smallest forms of animal existence, but it is now considered that a mite is twenty-saven million times larger than some of the animalcule disclosed by the microscope, some of these discernible in certain water belng so small that a thousand million of individuals, each with a distinct life of its own, occupy no more room than a grain of sand; but their number is sometimes so great that they give to the water they live in a red or yellow tinge. In this connection it is estimated that the milt of a codfish contains more minute animals than the whole human population of the world.

**Think a Moment**.—Statistics show that there is paid out in this country, in a single year:

| | |
|---|---|
| For Christian Home and Foreign Missions, per year, | $ 5,500,000 |
| For Public Education, | 85,000,000 |
| For Sugar and Molasses, | 155,000,000 |
| For Boots and Shoes, | 196,000,000 |
| For Cotton Goods, | 210,000,000 |
| For Sawed Lumber, | 233,000,000 |
| For Woolen Goods, | 237,000,000 |
| For Iron and Steel, | 290,000,000 |
| For Bread, | 505,000,000 |
| For Tobacco, | 600,000,000 |
| For Intoxicating Liquors, | 900,000,000 |

And the very persons who do most at this enormous expensive and purely injurious drink, are the ones that are constantly grumbling because they cannot get pay enough for their labor to enable them to live. How strange.

